# A Comprehensive Review of Platelet-Rich Plasma and Its Emerging Role in Accelerating Bone Healing

**DOI:** 10.7759/cureus.54122

**Published:** 2024-02-13

**Authors:** Milind R Gharpinde, Aditya Pundkar, Sandeep Shrivastava, Hardik Patel, Rohan Chandanwale

**Affiliations:** 1 Orthopedics, Jawaharlal Nehru Medical College, Datta Meghe Institute of Higher Education and Research, Wardha, IND

**Keywords:** accelerated fracture healing, growth factors, orthopedic surgery, regenerative medicine, bone healing, platelet rich plasma (prp)

## Abstract

This comprehensive review delves into the emerging role of platelet-rich plasma (PRP) in accelerating bone healing. PRP, a blood-derived product rich in platelets and growth factors, has garnered attention for its regenerative potential. The review begins by defining PRP and providing a historical background, highlighting its significance in expediting bone healing. PRP's composition and preparation methods, including centrifugation techniques and commercial kits, are explored. Mechanistically, PRP operates by releasing growth factors, chemotaxis, and angiogenesis, elucidating its cellular effects. Applications in fracture healing and orthopaedic surgeries, such as joint arthroplasty and spinal fusion, are discussed, emphasising the promising outcomes in clinical trials. Safety considerations, patient selection criteria, and the need for PRP preparation and application standardisation are underscored. The review outlines ongoing research trends, potential technological advancements, and unexplored areas in paediatric applications and inflammatory bone disorders. The implications for clinical practice involve informed decision-making, optimised protocols, and interdisciplinary collaboration. In conclusion, the future of PRP in bone healing holds exciting prospects, with the potential for precision medicine, integration with emerging therapies, expanded applications, and enhanced technological innovations shaping its trajectory in orthopaedics and regenerative medicine.

## Introduction and background

Platelet-rich plasma (PRP) is a blood-derived product that has gained significant attention in regenerative medicine due to its rich concentration of platelets and growth factors. PRP is obtained by centrifuging a patient's blood to separate and concentrate the platelets, which are then reintroduced into the body to stimulate tissue repair and regeneration. The increased concentration of platelets in PRP contains various bioactive proteins, including platelet-derived growth factor (PDGF), transforming growth factor-beta (TGF-β), and insulin-like growth factor (IGF), which play pivotal roles in the healing process [1]. The use of PRP in medical treatments traces its roots back to the 1970s, when it was initially employed in maxillofacial and oral surgery to enhance soft tissue healing. Over the years, advancements in medical research and technology have expanded the applications of PRP, particularly in orthopaedics and bone-related conditions. The historical progression of PRP reflects a growing understanding of its regenerative potential and has paved the way for its utilisation in various medical disciplines [2].

Bone healing is a complex and dynamic process that involves a sequence of events, including inflammation, cell proliferation, and tissue remodelling. The ability to accelerate bone healing is of paramount importance in the context of fractures, non-union fractures, and orthopaedic surgeries. Traditional approaches to enhance bone healing have limitations, and the emergence of PRP presents a promising alternative. By harnessing the body's natural healing mechanisms, PRP has shown the potential to expedite the healing process and improve outcomes in various bone-related conditions [3]. This review aims to provide a comprehensive overview of the emerging role of PRP in accelerating bone healing. This entails exploring PRP's composition and preparation methods, elucidating its mechanisms of action at the cellular level, and delving into its applications in fracture healing and orthopaedic surgeries. By critically examining the existing clinical evidence, safety considerations, and future research directions, this review aims to contribute to understanding PRP's efficacy and potential in enhancing bone regeneration.

## Review

Composition and preparation of PRP

Blood Components and Their Functions

Understanding blood composition is crucial for comprehending the rationale behind PRP therapy. Blood comprises various components, each playing a distinct role in the body's physiological processes. The key components include red blood cells (RBCs), white blood cells (WBCs), plasma, and platelets. While RBCs transport oxygen, WBCs are essential for immune response, and plasma is the liquid component carrying hormones, nutrients, and waste products. Platelets, however, are fundamental to hemostasis and wound healing. In PRP, the focus is on enriching and utilising the regenerative potential of platelets [4].

PRP Preparation Methods

Centrifugation techniques: Centrifugation is the predominant method for PRP preparation, leveraging the separation of blood components based on their density. The process begins with collecting a patient's blood, which is subsequently introduced into a centrifuge machine. The high-speed rotation of the centrifuge induces the separation of blood components into distinct layers. The platelet-rich layer is then extracted and collected for therapeutic use. Crucially, centrifugation speed and duration variations can yield different PRP formulations, such as leukocyte-rich or leukocyte-poor PRP. These formulations cater to specific bone-healing applications, providing versatility in addressing diverse clinical needs [5].

Buffy coat extraction: Buffy coat extraction offers an alternative approach to PRP preparation, utilising a density gradient for blood component separation. The blood is layered over a medium with varying density, and subsequent centrifugation forms distinct layers. The buffy coat, containing concentrated platelets and WBCs, is then harvested for therapeutic use. This method allows precise control over PRP composition, tailoring it to specific therapeutic requirements. Buffy coat extraction provides clinicians with flexibility in achieving desired platelet concentrations and cellular content, contributing to a personalised and targeted approach in bone healing applications [6].

Commercial PRP preparation kits: Commercial PRP preparation kits have gained widespread popularity for their convenience and standardisation. These kits offer a user-friendly and reproducible approach to PRP preparation by providing all the necessary components and detailed instructions. Typically, these kits incorporate specialised tubes and systems designed to optimise platelet concentration. While offering a streamlined and efficient method, it is crucial to consider factors such as kit-specific protocols and variations in achieved platelet concentrations. Clinicians benefit from the convenience of commercially available kits, appreciating the consistent and reliable outcomes they provide in preparing PRP for bone healing applications [7].

Mechanism of action

Release of Growth Factors

PDGF: PDGF stands out as a pivotal growth factor in platelets, significantly influencing cellular processes critical for bone healing. PDGF is central to stimulating cell proliferation, migration, and angiogenesis. PDGF recruits essential cells to the injury site in bone healing, including osteoblasts and mesenchymal stem cells (MSCs). The recruitment of these cells is paramount for initiating new bone tissue formation and the effective repair of fractures. PDGF's multifaceted effects create a regenerative microenvironment, emphasising its significance in the biological orchestration of bone healing processes [8].

TGF-β: TGF-β is a multifunctional cytokine with diverse roles in tissue repair and regeneration. In bone healing, TGF-β emerges as a key player in promoting the differentiation of MSCs into osteoblasts, the primary cells responsible for bone formation. Additionally, TGF-β enhances the production of extracellular matrix proteins, crucial for establishing the structural integrity of newly formed bone. TGF-β's dual impact on cell differentiation and matrix synthesis positions it as a fundamental growth factor in the cascade of events leading to effective bone regeneration. Its presence in PRP amplifies the regenerative potential of this biological therapy [9].

**Figure 1:**
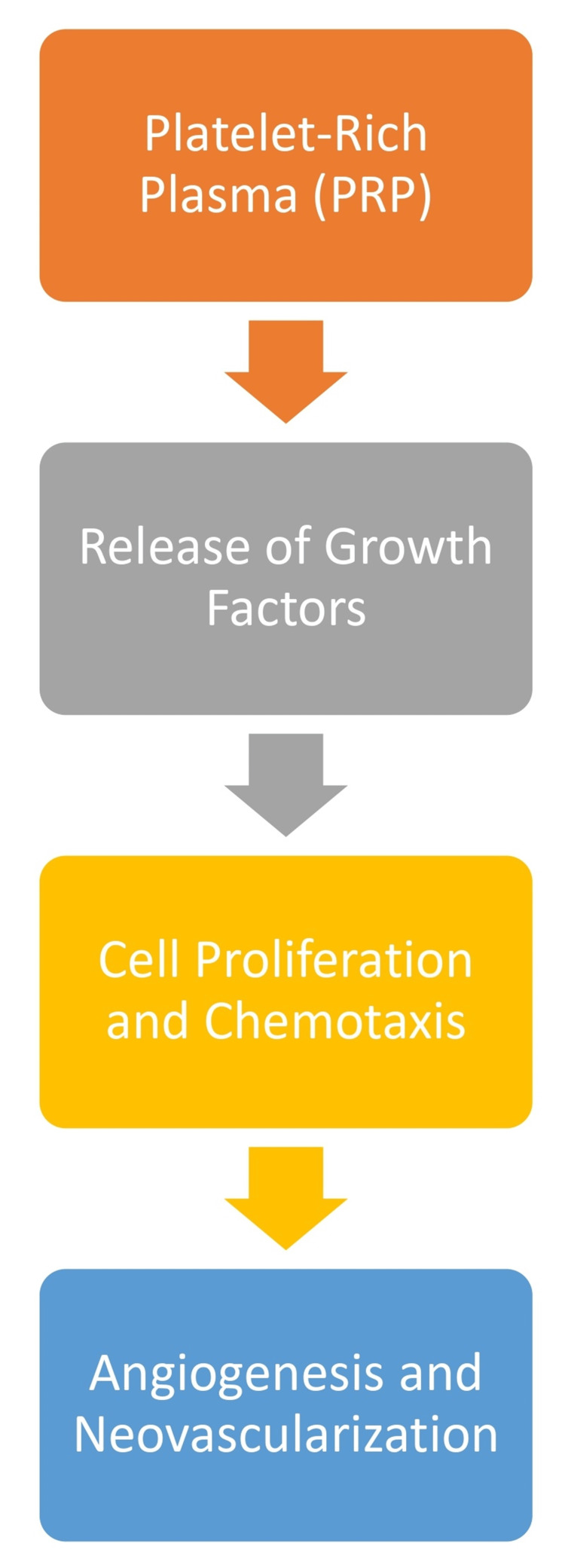
Mode of action of PRP in bone healing PRP: platelet-rich plasma

IGF: IGF is vital in promoting cell proliferation and differentiation, specifically for bone healing. Within bone regeneration, IGF contributes to the recruitment and differentiation of osteoblasts, pivotal cells in bone matrix synthesis. By facilitating the synthesis of bone matrix components, IGF supports the formation of structurally sound bone tissue. The collaborative action of PDGF, TGF-β, and IGF within the PRP milieu establishes a microenvironment conducive to efficient bone regeneration. The orchestrated interplay of these growth factors underscores their synergistic contribution to the regenerative potential of PRP in the context of bone healing [10]. The mode of action of PRP in bone healing is shown in Figure 1.

Chemotaxis and Cell Proliferation

Beyond the release of growth factors, PRP facilitates bone healing by promoting chemotaxis, the directed movement of cells towards the site of injury, and inducing cell proliferation. Chemotaxis involves recruiting various cell types to the injured area, including osteoblasts and MSCs. PRP enhances this process by providing a concentrated source of chemotactic factors, creating a gradient that guides cells to the site of bone damage. Cell proliferation is critical to bone healing and the formation of new bone tissue. PRP stimulates the proliferation of osteoblasts and MSCs, accelerating the production of bone matrix and supporting the overall regeneration process [11].

Angiogenesis and Neovascularisation

Angiogenesis, the intricate process of forming new blood vessels, plays a pivotal role in the multifaceted landscape of bone healing. PRP emerges as a key player in promoting angiogenesis, contributing to the restoration and regeneration of injured bone tissue. The mechanism through which PRP achieves this lies in its capacity to release angiogenic growth factors, initiating a cascade of events crucial for establishing an adequate blood supply to the injured area [12]. In bone healing, neovascularisation takes centre stage as a vital phenomenon. This process involves the creation of new blood vessels within the developing bone tissue, serving as a lifeline for nutrient delivery and waste removal. Neovascularisation is essential for sustaining the high metabolic demands associated with regenerating bone. PRP significantly contributes to this intricate process by fostering a well-vascularized network [13]. By releasing angiogenic growth factors, PRP creates a conducive environment for developing new blood vessels, ensuring that the regenerating bone receives nutrients and oxygen. This accelerates the healing process and enhances the robustness and resilience of the newly formed bone tissue. The pro-angiogenic properties of PRP underscore its significance in orchestrating the complex interplay of cellular and molecular events that characterise successful bone healing. PRP's promotion of angiogenesis represents a critical facet of its regenerative potential, aligning with the broader goal of optimising outcomes in orthopaedic and regenerative medicine contexts [14].

Applications in bone healing

Fracture Healing

Overview of the bone healing process: Fracture healing is a dynamic and intricate biological process crucial for restoring the integrity and functionality of damaged bones. This process unfolds in distinct stages, including inflammation, soft callus formation, complex callus formation, and remodelling. Each stage necessitates precise cellular interactions, orchestrated growth factor signalling, and extracellular matrix deposition. The coordinated sequence of events in fracture healing highlights the complexity of biological mechanisms, emphasising the need for targeted interventions that support and enhance these natural processes [13].

PRP in non-union fractures: Non-union fractures, marked by the failure of bones to heal within the expected timeframe, present a significant challenge in orthopaedics. PRP is a promising adjunctive therapy for non-union fractures. PRP addresses the biological factors that impede the natural healing process by providing a concentrated source of growth factors. Clinical studies exploring the effectiveness of PRP in non-union fractures have yielded encouraging results. PRP therapy promotes bone union, reduces the time to healing, and offers a targeted approach to address the complexities associated with non-union fractures. The regenerative properties of PRP contribute to creating a conducive environment for robust and accelerated bone healing in challenging clinical scenarios [15].

PRP in fresh fractures: In the context of fresh fractures, where immediate and robust healing is paramount, PRP has been investigated as a potential accelerant. The application of PRP at the fracture site aims to enhance the recruitment of osteogenic cells, stimulate the synthesis of bone matrix, and expedite the formation of a structurally sound callus. Studies exploring the use of PRP in conjunction with traditional fracture management have demonstrated promising outcomes. PRP therapy contributes to accelerated healing, improved biomechanical strength, and reduced recovery times in fresh fractures. This application underscores PRP's role as a regenerative intervention that complements standard fracture care, offering a pathway to enhance the efficiency and effectiveness of the natural bone healing process [16].

Orthopaedic Surgeries

Joint arthroplasty: Joint arthroplasty, encompassing procedures like total hip and total knee replacement, represents a critical intervention for individuals with damaged joint surfaces. The investigational use of PRP as an adjunctive therapy in joint arthroplasty aims to optimise postoperative healing and improve outcomes. PRP's potential to enhance tissue repair mechanisms and reduce inflammation is instrumental in fostering the early integration of prosthetic components. By contributing to the overall success of joint arthroplasty, PRP emerges as a promising avenue for augmenting the healing process and promoting improved functional outcomes in patients undergoing these intricate surgical procedures [17].

Spinal fusion: Spinal fusion, a surgical technique designed to stabilise the spine by encouraging the fusion of adjacent vertebral segments, has been enhanced through the investigational use of PRP. Acting as a biological enhancer, PRP's growth factors support the recruitment and differentiation of osteoblasts, critical cells involved in bone formation. This mechanism facilitates the development of a solid bony bridge between vertebral segments, potentially leading to improved fusion rates and enhanced clinical outcomes in spinal fusion surgeries. The application of PRP in this context underscores its role in advancing the biological processes critical for achieving successful spinal fusion and ensuring the long-term stability of the spine [18].

Osteotomy: Osteotomy, a surgical procedure involving the cutting and repositioning of bones to correct deformities or alter joint alignment, has benefited from investigations into the use of PRP. In osteotomy procedures, PRP has demonstrated its potential to accelerate bone healing and enhance the stability of surgical corrections. By promoting crucial processes such as cell proliferation, angiogenesis, and extracellular matrix synthesis, PRP contributes to expedited and robust bone union following osteotomy. This acceleration in the healing process reduces patients' recovery time. It introduces a valuable dimension to the precision and success of osteotomy procedures, positioning PRP as a supportive and regenerative adjunct in orthopaedic interventions [19].

Clinical evidence and studies

Review of Clinical Trials

PRP has been gaining popularity in various medical fields due to its potential to accelerate healing. PRP is a concentrated solution of platelets suspended in plasma obtained through the centrifugation of whole blood. Platelets play a crucial role in wound repair, and PRP delivers a supraphysiologic concentration of platelets to target tissues, harnessing their innate healing potential in a controlled manner [16,20]. In orthopaedic sports medicine, PRP has been used to treat tendon, ligament, muscle, and cartilage injuries and non-union fractures. Studies have reported that PRP may accelerate fracture healing by releasing growth factors important in the early stages of bone repair [19,21].

The clinical evidence supporting the use of PRP for bone fracture treatment is predominantly level IV, indicating a need for more randomised clinical trials. A systematic review by Zhang et al. reported that PRP shortened bone healing duration and positively improved the healing rate of closed fractures [16]. A randomised controlled trial by Ghaffarpasand et al. found that PRP treatment increased the healing rate of long bone non-union fractures compared to the control group [19]. A systematic review and network analysis by Li et al. found that PRP was a promising alternative treatment for patients with long-bone delayed union and non-union, with a healing rate of 85.80% in the PRP group compared to 60.76% in the control group [21]. Despite these promising results, PRP treatment's long-term adverse side and functional outcome still need further large-scale trials and long-term follow-up [21].

Limitations and Challenges in the Existing Research

Controversial findings: The clinical landscape surrounding PRP for bone fracture treatment is marked by a spectrum of findings, often controversial and contradictory. While certain studies assert that PRP accelerates bony healing, reducing the duration of the healing process, others present contrasting results, indicating no discernible positive effect on the healing rate, particularly in the context of closed fractures [16]. This divergence in findings underscores the complexity of PRP's role in bone healing. It highlights the need for a nuanced understanding of the specific conditions, fracture types, and patient characteristics that may influence the outcomes. The presence of controversial findings underscores the ongoing challenges in establishing a consensus regarding the unequivocal benefits of PRP in bone fracture treatment.

Variability in preparation protocols: A significant impediment to comprehensively evaluating the clinical effects of PRP lies in the variability of established preparation protocols. The existence of numerous commercial systems designed for PRP preparation contributes to a diverse array of PRP products and introduces potential variability in patient outcomes [22]. This lack of standardisation in preparation protocols hinders comparing and generalising results across studies. Understanding the impact of these protocol variations is crucial for interpreting conflicting findings and underscores the necessity for standardized approaches to PRP preparation in bone healing research.

Lack of standardisation: One of the overarching challenges in the field of PRP for bone healing is the absence of univocal guidelines for PRP preparation. The molecular mechanisms underlying PRP's actions in bone healing have yet to be fully elucidated, further complicating the standardisation of preparation protocols [23]. The lack of a standardised approach contributes to the variability in PRP formulations and poses challenges in interpreting and replicating research findings. Achieving consensus on standardised preparation guidelines is imperative for advancing the understanding of PRP's role in bone healing and establishing a foundation for future research and clinical applications.

Need for more randomized controlled trials (RCTs): The current clinical evidence supporting the use of PRP for bone fracture treatment is predominantly categorized as level IV, indicating a reliance on lower levels of evidence, such as case series and observational studies. This highlights a pressing need for more RCTs to conclusively establish the efficacy and safety of PRP in bone fracture treatment [16,19]. RCTs provide a higher level of evidence, offering a more robust foundation for evidence-based practice. Conducting well-designed RCTs that adhere to standardised protocols is essential for addressing the existing controversies, enhancing the reliability of research outcomes, and informing clinical decision-making regarding the use of PRP in bone fracture treatment.

Long-term adverse side effects and functional outcomes: Despite the promising results observed in certain studies, exploring PRP treatment for bone fractures emphasises the imperative for further investigation into its long-term adverse side effects and functional outcomes. Large-scale trials and extended follow-up periods are crucial to comprehensively assess the safety profile of PRP and ascertain its impact on functional outcomes over the long term [22]. This recognition underscores the importance of short-term efficacy and PRP's sustained safety and functional benefits in bone fracture treatment, ensuring a comprehensive understanding of its impact on patient outcomes.

Variability in patient characteristics: Another layer of complexity in evaluating the outcomes of PRP treatment for bone fractures arises from the variability in patient characteristics. Factors such as age, medical comorbidities, and individual healing capabilities introduce additional variation in PRP products, potentially contributing to divergent findings in the existing literature [22]. Acknowledging and understanding these patient-specific differences is paramount for contextualising research outcomes and tailoring PRP treatment approaches to individualised patient needs. This consideration highlights the importance of a personalised medicine approach in applying PRP for bone fracture treatment.

Need for further research: While some studies have reported positive effects of PRP on bone healing, the call for further research remains imperative to address existing limitations and challenges. These challenges include the need for more standardised preparation protocols, expanded RCTs, and long-term follow-up assessments to comprehensively evaluate the safety and efficacy of PRP for bone fracture treatment. The identified gaps underscore the ongoing evolution of PRP research, emphasising the necessity for methodologically rigorous studies that address the complexities associated with PRP treatment. More extensive research efforts will contribute to refining protocols, enhancing the evidence base, and ultimately guiding the integration of PRP into mainstream clinical practice for bone fracture management.

Safety and considerations

Adverse Effects and Complications

Infection: The risk of infection at the injection site is a paramount consideration in PRP therapy. Mitigating this risk necessitates strict adherence to aseptic techniques throughout PRP preparation and administration. Rigorous hygiene protocols safeguard patient well-being and uphold the integrity of the therapeutic intervention. By prioritising infection prevention measures, healthcare providers can ensure the safety and efficacy of PRP treatments, minimise the potential for complications, and promote positive patient outcomes [24].

Pain and discomfort: Acknowledging the possibility of temporary pain or discomfort at the injection site is a crucial aspect of patient care during and following PRP therapy. While such sensations are typically mild and transient, providing patients with clear expectations regarding potential discomfort is essential. This proactive communication allows for informed consent and empowers patients to manage post-treatment experiences effectively. Clinicians can implement supportive measures to alleviate discomfort, enhance the overall patient experience, and foster a positive perception of PRP therapy [25].

Allergic reactions: Although rare, the prospect of allergic reactions to components present in PRP necessitates careful screening and monitoring. Before initiating PRP therapy, thorough allergy assessments contribute to preemptive risk management. Additionally, vigilant monitoring for any signs of allergic reactions during and after the procedure ensures timely intervention if such rare events occur. This attention to allergic considerations underscores the commitment to patient safety and the importance of personalised care in PRP therapy [26].

Excessive inflammation: The potential for PRP to induce excessive inflammation introduces a nuanced consideration in its therapeutic application. While inflammation is a natural part of the healing process, its excessive manifestation could hinder rather than promote healing. Monitoring for signs of disproportionate inflammatory responses is crucial, enabling clinicians to tailor treatment plans accordingly. This awareness emphasises the need for a balanced and nuanced approach to PRP therapy, aligning treatment outcomes with the intended regenerative effects and minimising unintended complications [27].

Nerve or blood vessel damage: The risk of nerve or blood vessel damage underscores the importance of precise administration and placement of PRP injections. Utilising imaging guidance and carefully selecting injection sites are critical strategies to minimise this risk. By ensuring accurate delivery of PRP to the intended tissues while avoiding sensitive structures, healthcare providers can enhance the safety profile of PRP therapy. This meticulous approach aligns with the principle of minimising procedural risks, contributing to overall patient safety, and optimising the therapeutic impact of PRP in bone healing [28].

Patient Selection Criteria

The severity of the condition: Tailoring the application of PRP based on the severity of the condition represents a strategic approach to optimising therapeutic outcomes. PRP may exhibit enhanced efficacy in non-union fractures or orthopaedic procedures where accelerated healing is imperative. This consideration acknowledges the nuanced nature of bone injuries and conditions, ensuring that PRP is judiciously applied where its regenerative potential can have the most significant impact. Assessing the severity of the condition serves as a guiding principle in determining the appropriateness of PRP therapy and aligning treatment strategies with each patient's specific needs [29].

General health status: Patients' general health status is a crucial factor requiring careful evaluation before PRP therapy. Underlying health conditions, such as diabetes, autoimmune disorders, or infections, may influence the efficacy of PRP. Considering the patient's overall health provides a comprehensive understanding of the physiological context in which PRP will function. This evaluation ensures that PRP therapy is administered with due consideration for potential interactions or limitations posed by the patient's health status, contributing to a more tailored and patient-centred approach [30].

Age: While PRP has demonstrated effectiveness across various age groups, the regenerative capacity of tissues may exhibit variability based on age. Recognising that older patients may experience a slower healing response underscores the importance of age as a patient-specific factor. This consideration informs clinicians about potential variations in treatment outcomes, allowing for adjustments in treatment plans and setting realistic expectations based on the patient's age-related healing dynamics [31].

Contraindications: Patients with bleeding disorders or those taking anticoagulant medications present specific contraindications to PRP therapy due to the heightened risk of increased bleeding. This safety consideration emphasises the necessity of thorough patient screening and highlights the importance of identifying contraindications before initiating PRP treatments. Ensuring patient safety by adhering to contraindications aligns with best practices and mitigates potential risks associated with PRP therapy [32].

Patient expectations: Clear and comprehensive communication with patients regarding the expected outcomes, potential risks, and the experimental nature of PRP therapy is paramount. Managing patient expectations establishes a transparent foundation for the therapeutic journey, fosters informed decision-making, and enhances the patient's understanding of the treatment process. This communication is instrumental in building trust between clinicians and patients, empowering individuals to actively participate in their care and make well-informed choices regarding PRP therapy [33].

Standardisation of PRP Preparation and Application

Preparation protocols: Establishing standardised protocols for PRP preparation stands as a cornerstone in ensuring the reproducibility and reliability of PRP formulations. Defining specific parameters such as centrifugation speeds, timings, and the use of anticoagulants is pivotal for generating consistent PRP products across different settings. These protocols provide a framework for practitioners to follow and contribute to the scientific rigour of PRP research and clinical applications. Standardisation in preparation protocols is fundamental for comparing outcomes across studies and refining treatment approaches based on evidence-based practices [34].

Quality control: Regular monitoring and verification of the quality of PRP preparations are essential components in maintaining the integrity and consistency of PRP formulations. This includes assessing platelet concentration, purity, and other relevant parameters. Quality control measures ensure that PRP products meet predefined standards, mitigating variability and enhancing the reliability of therapeutic outcomes. Incorporating stringent quality control practices into PRP preparation protocols is crucial for upholding safety standards, meeting regulatory requirements, and fostering confidence among clinicians and patients in the therapeutic potential of PRP [1].

Injection techniques: Standardising injection techniques for PRP administration is paramount to ensuring uniform delivery of PRP to target tissues. This involves specifying the depth and location of injections, factors that can significantly influence the therapeutic impact of PRP. Consistency in injection techniques not only enhances the precision and efficacy of PRP therapy but also minimises the risk of variations in treatment outcomes. Standardisation in injection techniques is integral to promoting best practices, reducing the likelihood of procedural errors, and optimising the therapeutic benefits of PRP across diverse clinical applications [35].

Follow-up protocols: Implementing standardised follow-up protocols is crucial for monitoring patient outcomes, detecting potential adverse effects, and making informed adjustments to treatment plans. Regular and systematic follow-up contributes to ongoing safety and efficacy assessments, providing valuable data on the long-term impact of PRP therapy. Standardised follow-up protocols enable clinicians to refine treatment strategies based on patient responses, fostering a dynamic and evidence-driven approach to PRP applications. This iterative feedback loop ensures that PRP treatments remain patient-centred, adaptive, and aligned with the evolving understanding of PRP's role in bone healing and regenerative medicine [36].

Future directions and research needs

Ongoing Research and Emerging Trends

Cellular and molecular mechanisms: Exploring the cellular and molecular mechanisms underlying PRP's effects on bone healing constitutes a critical frontier in research. Unravelling the intricate signalling pathways involved in PRP's regenerative actions can provide invaluable insights into its mode of action. This deeper understanding is pivotal for refining and optimising PRP formulations to be more targeted and effective. By elucidating the specific cellular responses and molecular interactions triggered by PRP, researchers can tailor formulations to enhance desired outcomes, ultimately advancing the precision and efficacy of PRP therapy in bone healing [37].

Combination therapies: Investigating the potential synergies between PRP and other regenerative therapies, such as stem cell therapies or biomaterials, is promising for advancing bone regeneration strategies. Combining PRP with complementary approaches can create a more comprehensive and potent regenerative effect. Understanding the synergistic interactions between PRP and other therapeutic modalities can guide the development of innovative and integrated treatment protocols, offering a multifaceted approach to address the complex challenges associated with diverse bone injuries and conditions [38].

Dose-response relationships: Determining the optimal dosage and concentration of PRP for different applications is a critical aspect that requires ongoing research efforts. Establishing clear dose-response relationships is crucial for refining treatment protocols and ensuring that PRP is administered at levels that maximise therapeutic efficacy. This research endeavour is fundamental for standardising PRP applications in diverse clinical scenarios, allowing clinicians to tailor treatments based on the specific needs of individual patients and the nature of their bone injuries [39].

Long-term outcomes: Comprehensive studies with extended follow-up periods are imperative to assess the long-term outcomes of PRP therapy in bone healing. Evaluating the sustainability of improvements, identifying potential late-onset complications, and determining the overall durability of the regenerative effects are essential considerations. Long-term outcome research not only provides insights into the lasting impact of PRP therapy but also contributes valuable data for establishing the safety and efficacy profile of PRP over extended periods. This knowledge is paramount for informing clinical practice and shaping the evolving landscape of PRP applications in bone healing [40].

Potential Advancements in PRP Technology

Customised formulations: As PRP continues to gain traction in bone healing, the prospect of customised formulations represents a significant technological advancement. Tailoring PRP formulations to specific patient characteristics or types of bone injuries acknowledges the inherent variability in healing capacity among individuals. By personalising PRP compositions, this approach can potentially optimise treatment outcomes, ensuring patients receive therapy that suits their unique needs. This individualised strategy aligns with the broader medical trend towards personalised and precision approaches, offering a more nuanced and targeted therapeutic solution [41].

Biocompatible carriers: The development of biocompatible carriers or scaffolds is a promising avenue for enhancing the delivery and retention of PRP at injury sites. These carriers, designed to be compatible with the body's biological environment, could serve as supportive frameworks for the sustained release of growth factors present in PRP. By providing a conducive environment for cellular activities and facilitating the controlled release of therapeutic components, biocompatible carriers aim to improve the overall efficacy of PRP therapy. This innovation can potentially enhance treatment outcomes, especially in cases where the targeted and controlled release of growth factors is crucial for effective bone regeneration [42].

Innovations in application techniques: Advancements in delivery methods for PRP, such as minimally invasive procedures or guided injection techniques, mark a significant stride in improving the precision and effectiveness of PRP applications. These innovations aim to enhance the therapeutic impact while minimising invasiveness by refining how PRP is delivered to the target site. Improved delivery methods have the potential to facilitate more targeted treatment, ensuring that PRP reaches the intended areas with greater accuracy. Additionally, these advancements can reduce the risk of complications, further establishing PRP as a safe and reliable option for bone healing [43].

Point-of-care devices: The development of point-of-care devices represents a transformative leap in the administration of PRP therapy. These devices, designed for rapid and on-site preparation of PRP, have the potential to streamline the treatment process significantly. By eliminating the need for off-site processing, point-of-care devices enhance accessibility and convenience for both clinicians and patients. This innovation aligns with the growing emphasis on patient-centred care, offering a more efficient and immediate approach to PRP therapy. As point-of-care technology evolves, it promises to make PRP therapy a more accessible and widely applicable solution in various clinical settings [44].

Unexplored Areas in Bone Healing and PRP

Paediatric applications: Limited research exists on using PRP in paediatric orthopaedics, representing an essential avenue for exploration. The safety and efficacy of PRP in promoting bone healing in children have yet to be extensively studied. Investigating this application could contribute to understanding how PRP impacts paediatric bones' unique physiological and developmental aspects. Addressing this knowledge gap is crucial for optimising treatment approaches in the paediatric population, where bone injuries and conditions are distinct from those in adults [45].

Systemic effects: Exploring the potential systemic effects of PRP beyond its local application site marks an uncharted area in research. While the focus has traditionally been on localised regenerative effects, understanding how PRP may influence systemic factors involved in bone metabolism is a critical frontier. This broader perspective could unveil systemic benefits or implications of PRP therapy, offering insights into its holistic impact on the musculoskeletal system and overall bone health [46].

Inflammatory bone disorders: The application of PRP in inflammatory bone disorders, such as osteomyelitis or rheumatoid arthritis, remains relatively unexplored. In these conditions characterised by chronic inflammation and bone degeneration, PRP's potential to modulate the inflammatory milieu and promote regenerative processes could open new therapeutic possibilities. Research in these areas holds promise for uncovering novel treatment approaches and addressing the complex challenges associated with inflammatory bone disorders [47].

Patient-specific factors: Tailoring PRP treatments based on patient-specific factors represents a personalised approach that has yet to be extensively investigated. Genetic variations or comorbidities may influence how individuals respond to PRP therapy. Delving into the interplay between patient characteristics and PRP response can guide the development of personalised regenerative approaches, optimising treatment outcomes, and pave the way for more effective and targeted interventions. This avenue of research aligns with the broader medical trend towards precision and personalised medicine [27].

## Conclusions

Exploring PRP in bone healing presents a multifaceted approach to regenerative medicine. The review has elucidated the intricate mechanisms through which PRP accelerates bone healing, emphasising the release of growth factors, chemotaxis, and angiogenesis. Its applications in fracture healing, various orthopaedic surgeries, safety considerations, and patient selection criteria underscore its potential as a valuable therapeutic option. The implications for clinical practice emphasise the importance of informed decision-making, optimised protocols, and interdisciplinary collaboration. Looking ahead, the future of PRP in bone healing holds promise for precision medicine, integration with emerging therapies, expanded applications in unexplored areas, and enhanced technological advancements. As the field evolves, the potential for PRP to play a significant role in improving patient outcomes in bone healing remains an exciting and dynamic area of exploration, with ongoing research and innovation shaping its trajectory.
